# Supplementary material for: OsPHR2-mediated recruitment of Pseudomonadaceae enhances rice phosphorus uptake
Source: Plant Commun. 2024 Apr 29;5(8):100930. doi: 10.1016/j.xplc.2024.100930 (PMC11369732; doi:10.1016/j.xplc.2024.100930)
Supplement: Document S1. Supplemental Table 1 and Supplemental Figures 1–7 [file mmc1.pdf]

Plant Communications, Volume *5*

Supplemental information

OsPHR2-mediated recruitment of Pseudomonadaceae enhances rice phosphorus uptake

Jianping Liu, Weifeng Xu, Qian Zhang, Wencheng Liao, Liang Li, Shu Chen, Jinyong Yang, Zhengrui Wang, and Feiyun Xu

1. Supplementary Tables

Table S1 Soil chemical factors.

Soil component	Organic C (g/kg)	Total N (g/kg)	Total P (g/kg)	Total K (g/kg)	Olsen-P (mg/kg)	Exchangeable K (mg/kg)
	35.6	1.02	0.537	36.3	49.5	92

2. **Supplementary Figures**

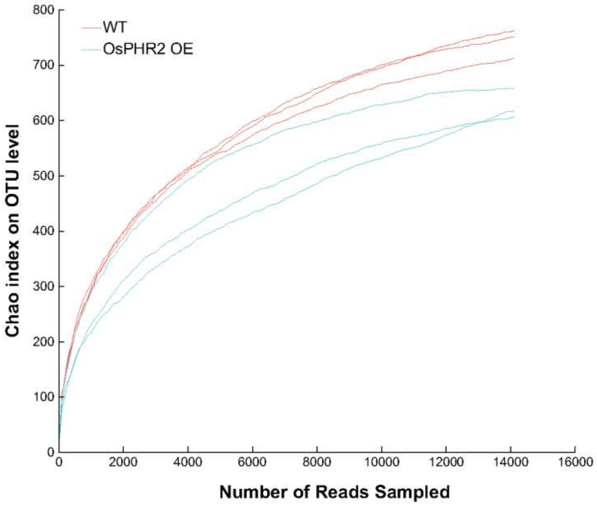

Fig. S1 The gradually flattening rarefaction curves of root samples.

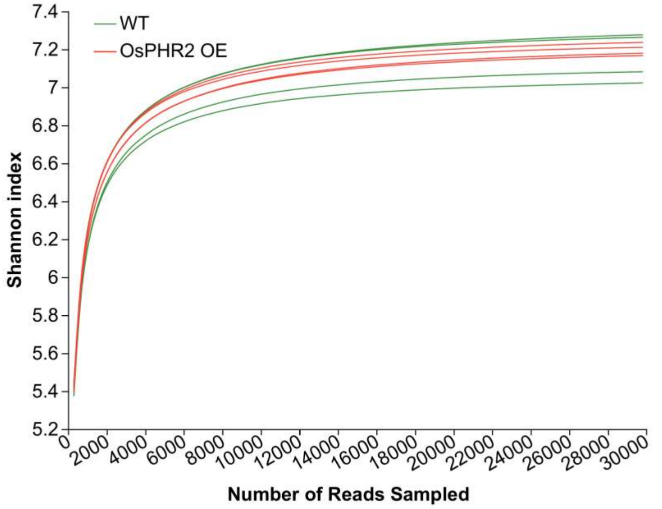

Fig. S2 The gradually flattening rarefaction curves of rhizosphere soil samples.

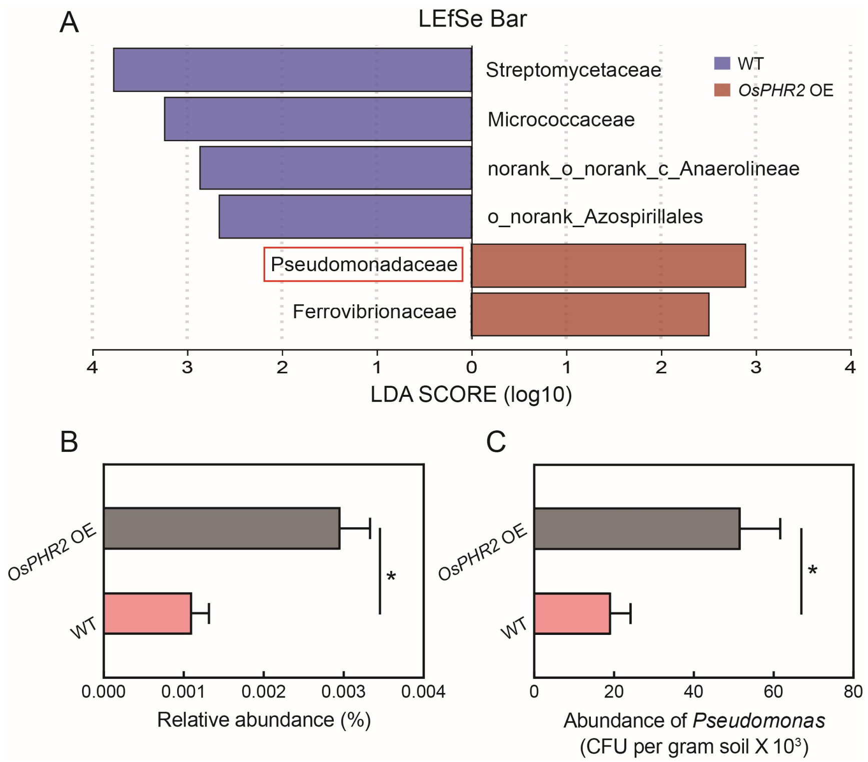

Fig. S3 A, Liner discriminant analysis effect size (LEfSe) analysis of bacterial taxon with significant difference in abundances in WT and *OsPHR2* OE rhizosphere soil. Linear discriminant analysis score ≥ 2.5. **B,** Relative abundances of Pseudomonadaceae in WT and *OsPHR2* OE rhizosphere soil. Data are means ± SD (n = 3). **C,** Abundance of cultivable *Pseudomonas* in rhizosphere soil of WT and *OsPHR2* OE rice. Data are means ± SD (n = 3). Asterisks indicate significant difference between soil environments by two-sided Student's t-test (*$p < 0.05$).

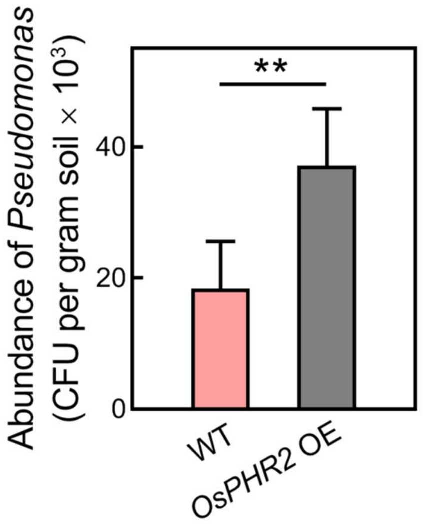

Fig. S4 Abundance of cultivable *Pseudomonas* in root of WT and *OsPHR2* OE rice in low phosphorus soil. Data are means ± SD (n = 6). Asterisks indicate significant difference between soil environments by two-sided Student's t-test (*p < 0.01).

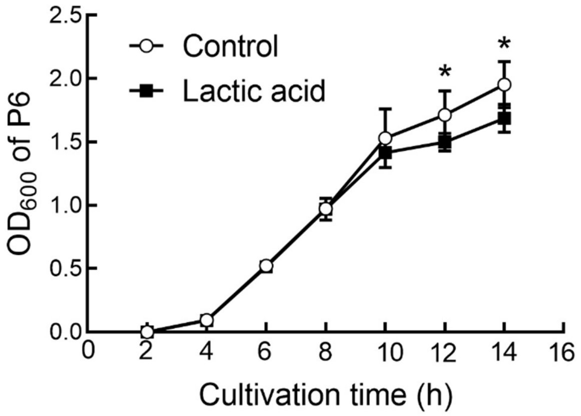

Fig. S5 The growth of strain P6 with addition of lactic aicd. Data are means ± SD (n = 5). Asterisks indicate significant difference by two-sided Student's t-test (*$p < 0.05$).

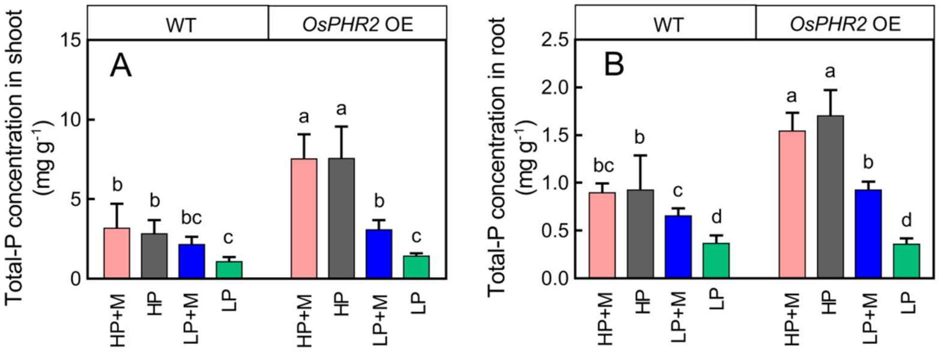

Fig. S6 Shoot and root total P concentration of WT and *OsPHR2* OE rice inoculated with mixed strains under HP (HP+M), not inoculated under HP (HP), inoculated with mixed strains under LP (LP+M), not inoculated under LP (LP). Data are means ± SD (n = 5). Bars with different letters among different treatments indicate significantly different at $p < 0.05$ (ANOVA, Duncan's multiple range test).

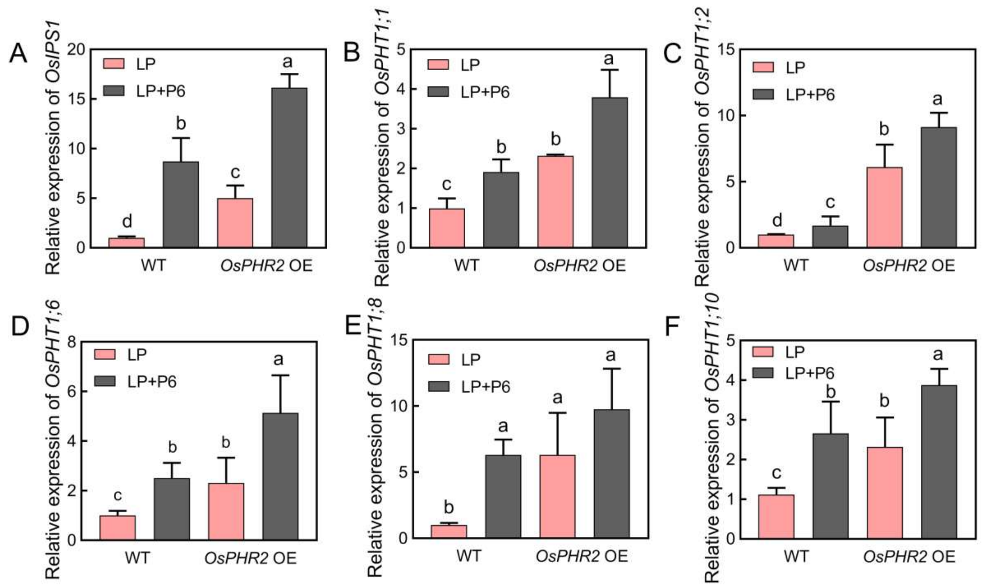

Fig. S7 Relative expression of P transporter genes and P starvation-induced marker gene in WT and *OsPHR2* OE rice inoculated with P6 under LP (LP+P6), not inoculated under LP (LP). Data are means ± SD (n = 3). Bars with different letters among different treatments indicate significantly different at $p < 0.05$ (ANOVA, Duncan's multiple range test).
