# Supplementary material for: OsPHR2-mediated recruitment of Pseudomonadaceae enhances rice phosphorus uptake
Source: Plant Commun. 2024 Apr 29;5(8):100930. doi: 10.1016/j.xplc.2024.100930 (PMC11369732; doi:10.1016/j.xplc.2024.100930)
Supplement: Document S2. Article plus supplemental information [file mmc2.pdf]

Plant Communications
Research article

OsPHR2-mediated recruitment of Pseudomonadaceae enhances rice phosphorus uptake

Jianping Liu[1,2], Weifeng Xu[1,2,*], Qian Zhang[1,2], Wencheng Liao[1], Liang Li[1], Shu Chen[1], Jinyong Yang[1], Zhengrui Wang[1] and Feiyun Xu[1,*]

[1]Center for Plant Water-use and Nutrition Regulation and College of JunCao Science and Ecology, Joint International Research Laboratory of Water and Nutrient in Crop, Fujian Agriculture and Forestry University, Fuzhou 350002, China

[2]These authors contributed equally.

***Correspondence: Weifeng Xu (wfxu@fafu.edu.cn), Feiyun Xu (xufy@fafu.edu.cn)**

https://doi.org/10.1016/j.xplc.2024.100930

ABSTRACT

Plants can shape their root microbiome to promote growth and nutrient uptake. PHOSPHATE STARVATION RESPONSE 2 (OsPHR2) is a central regulator of phosphate signaling in rice, but whether OsPHR2 can shape the root microbiome to promote phosphorus uptake is unclear. Here, we investigate the role of OsPHR2 in recruiting microbiota for phosphorus uptake using high-throughput sequencing and metabolite analysis. *OsPHR2*-overexpressing (*OsPHR2* OE) rice showed 69.8% greater shoot P uptake in natural soil compared with sterilized soil under high-phosphorus (HP) conditions, but there was only a 54.8% increase in the wild-type (WT). The abundance of the family Pseudomonadaceae was significantly enriched in *OsPHR2* OE roots relative to those of WT rice. Compared with the WT, *OsPHR2* OE rice had a relatively higher abundance of succinic acid and methylmalonic acid, which could stimulate the growth of *Pseudomonas sp. (P6)*. After inoculation with P6, phosphorus uptake in WT and *OsPHR2* OE rice was higher than that in uninoculated rice under low-phosphorus (LP) conditions. Taken together, our results suggest that *OsPHR2* can increase phosphorus use in rice through root exudate-mediated recruitment of *Pseudomonas*. This finding reveals a cooperative contribution of the OsPHR2-modulated root microbiome, which is important for improving phosphorus use in agriculture.

Keywords: OsPHR2, rice, organic acid, phosphorus, Pseudomonadaceae

Liu J., Xu W., Zhang Q., Liao W., Li L., Chen S., Yang J., Wang Z., and Xu F. (2024). OsPHR2-mediated recruitment of Pseudomonadaceae enhances rice phosphorus uptake. Plant Comm. **5**, 100930.

INTRODUCTION

Phosphorus (P), a non-renewable resource, is an essential element for plant growth and participates in many metabolic pathways (Rubio et al., 2001). However, P fertilizer application is inefficient, as only 10%–25% of applied P is taken up by plants (Johnston et al., 2014). Limited soil P is typically available for plant growth, owing to its formation of insoluble complexes with Ca^{2+}, Al^{3+}, and Fe^{3+}, and about 70% of global arable land is P deficient, compromising plant growth and crop yields. To increase crop yields, large quantities of P fertilizers are applied in the field, which leads to depletion of global P reserves (Gilbert, 2009). Therefore, exploration of the regulatory mechanisms that underlie P uptake is essential for improvement of crop P acquisition.

Bacteria play a key role in overcoming the challenges of P-deficient soil (Lidbury et al., 2020). Some bacteria are able to solubilize phosphate (Mosimann et al., 2017), which may be essential in P-deficient agricultural soils. Phosphate-solubilizing bacteria can increase plant yield by enhancing plant P-acquisition strategies and P distribution (Raymond et al., 2021). Members of the phyla *Firmicutes*, *Actinobacteria*, and *Proteobacteria* are currently known as phosphate-solubilizing bacteria (Liang et al., 2020). At the genus level, strains isolated from the genera *Pseudomonas*, *Bacillus*, and *Rhizobium* have generally shown the greatest P-solubilizing ability (Alori et al., 2017). *Streptomyces griseorubens* BC3 and *Norcardiopsis alba* BC11 significantly increased root and shoot P content and grain yield in maize (Soumare et al., 2021). In addition,

Published by the Plant Communications Shanghai Editorial Office in association with Cell Press, an imprint of Elsevier Inc., on behalf of CSPB and CEMPS, CAS.

This is an open access article under the CC BY license (http://creativecommons.org/licenses/by/4.0/).

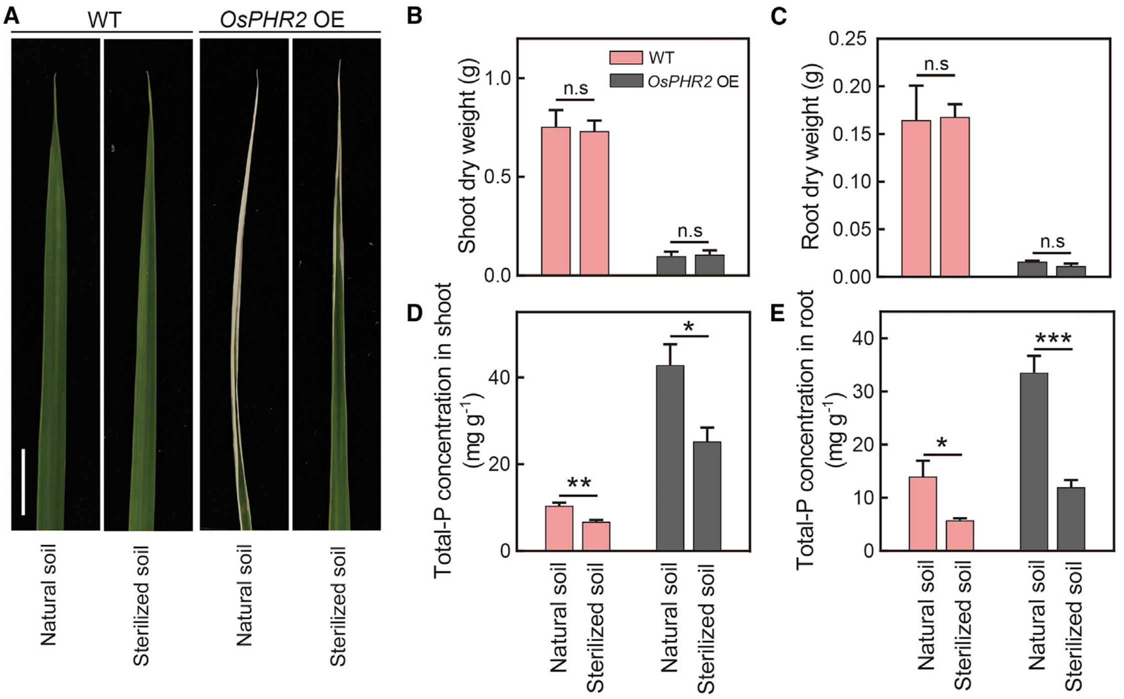

Figure 1. The microbiota is involved in the promotion of phosphorus uptake by PHR2 in rice.
(A) Necrosis of old leaf blade tips in WT rice and the *OsPHR2*-overexpressing line (*OsPHR2* OE) under high-P (HP) conditions. Bar: 1 cm.
(B and C) Shoot and root dry weights of WT and *OsPHR2* OE rice. Data are means ± SD ($n = 5$).
(D and E) Total P concentrations in shoots and roots of WT and *OsPHR2* OE rice. Data are means ± SD ($n = 5$).
Asterisks indicate significant differences among treatments determined by two-sided Student's *t*-test (*$p < 0.05$, **$p < 0.01$, and ***$p < 0.001$). n.s., not significant. See also Supplemental Table 1.

inoculation with phosphate-solubilizing bacteria can enable a 50% reduction in P fertilizer application without a decrease in crop yield (Rafi et al., 2019). In chickpea, inoculation with *Pseudomonas jessenii* significantly enhanced shoot dry weight compared with that of uninoculated plants (Valverde et al., 2006).

Recent studies suggest that the P starvation response system has an important role in regulating soil microbes for solubilization of insoluble P (Castrillo et al., 2017; Isidra-Arellano et al., 2021; Tang et al., 2022), and PHOSPHATE STARVATION RESPONSE (PHR) proteins play central parts in this system. They are important for modulating P starvation responses through binding to the *cis* element P1BS in the promoters of P-starvation-induced genes (Zhou et al., 2008; Wang et al., 2013; Ruan et al., 2017). Rice overexpressing *OsPHR2* displayed excessive P accumulation in leaves, which resulted in P toxicity (Zhou et al., 2008). Under low-P (LP) stress, *AtPHR1* can regulate the expression of P-starvation-induced genes such as *AtIPS1*, *AtRNS1*, and *At4* (Raghothama, 1999). PHR proteins can also regulate P uptake from external sources in different tissues by binding to the promoters of PHOSPHATE TRANSPORTER genes (Liu et al., 2010) and can modulate the expression of *miRNA399* and *miRNA827* to regulate P homeostasis and signaling (Miura et al., 2005; Kant et al., 2011; Lin et al., 2013). In addition to their regulatory functions in plants, PHR proteins can also interact with microbiota to alleviate P starvation. For example, *Arabidopsis PHR1* can shape root microbiome communities (Castrillo et al., 2017). PHR-centered networks can control arbuscular mycorrhizal symbioses, and overexpression of *OsPHR2* improves mycorrhizal infection in rice (Shi et al., 2021). Immunity suppression by the PHR1–RALF–FERONIA module enhances root bacterial growth, which can increase the expression of PHR genes to enhance plant P uptake (Tang et al., 2022). However, it is unclear whether OsPHR2 can enable recruitment of bacteria to promote rice P uptake.

In the present study, we investigated the role of *OsPHR2* in P uptake using wild-type (WT) and *OsPHR2*-overexpressing (*OsPHR2* OE) rice. rRNA gene amplicon sequencing of rice roots from high-P (HP) conditions was performed to identify bacteria involved in P uptake. A number of candidate strains were isolated and their ability to solubilize phosphate was assessed. We tested the effects of a phosphate-solubilizing strain on rice P uptake using WT and *OsPHR2* OE rice grown under HP and LP conditions. Our findings provide evidence for the promotion of rice P uptake by root microbiota.

RESULTS

Microbiota associated with phosphorus uptake in *OsPHR2* OE rice under HP conditions

To investigate whether the microbiota is associated with *OsPHR2*-regulated P uptake in rice, rice plants were grown in natural and sterilized soils (Figure 1A). The P toxicity phenotype observed in *OsPHR2* OE rice grown on natural soil was alleviated by growth on sterilized soil (Figure 1A). By contrast, WT rice showed no necrotic phenotype on either natural or

sterilized soil (Figure 1A). Under HP conditions, shoot and root dry weights of *OsPHR2* OE rice did not differ significantly between natural soil and sterilized soil (Figure 1B), and the same trend was observed for WT rice (Figure 1C). However, *OsPHR2* OE rice exhibited 69.8% greater shoot P uptake and 179.9% greater root P uptake in natural soil than in sterilized soil under HP conditions (Figure 1D and 1E). WT plants exhibited only 54.8% greater shoot P uptake and 143.0% greater root P uptake in natural soil than in sterilized soil. These results suggested that microbiota might be associated with phosphorus uptake in *OsPHR2* OE rice.

Rice root and rhizosphere bacterial composition in WT and *OsPHR2* OE rice

To explore OsPHR2-induced changes in root and rhizosphere bacteria, we built 16S rRNA amplicon libraries for the roots and rhizospheres of WT and *OsPHR2* OE rice under HP conditions (Figure 2). The sequencing depth adequately captured the complete microbial communities, as evidenced by gradual flattening of the rarefaction curves (Supplemental Figures 1 and 2). The dominant phyla in the roots of WT and *OsPHR2* OE rice under HP conditions included Proteobacteria, Actinobacteria, Myxococcota, Chloroflexi, Fibrobacterota, and Bacteroidota (Figure 2A). At the family level, Comamonadaceae (relative abundance: 51.7%) were significantly more abundant in *OsPHR2* OE roots than in WT roots under HP conditions (Figure 2B). To further investigate the root-dependent microbiota of WT and *OsPHR2* OE rice under HP conditions, we performed linear discriminant analysis (LDA) effect size (LEfSe) analysis to evaluate bacterial biomarkers in the root microbiome (Figure 2C). In the root samples, Pseudomonadaceae (LDA score: 4.02) was specifically enriched in *OsPHR2* OE rice under HP conditions (Figure 2C). In the rhizosphere samples, Pseudomonadaceae (LDA score: 2.89) was again specifically enriched in *OsPHR2* OE rice (Supplemental Figure 3A). The abundance of family Pseudomonadaceae was increased 26.1-fold in *OsPHR2* OE rice compared with WT rice (Figure 2D). Colony counting was used to confirm the effect of OsPHR2 on Pseudomonadaceae abundance, and the abundance of culturable *Pseudomonas* was 124% higher in *OsPHR2* OE roots than in WT roots (Figure 2E). In the rhizosphere, Pseudomonadaceae abundance was increased by 168% in *OsPHR2* OE rice compared with WT rice (Supplemental Figure 3B), and colony counting of culturable *Pseudomonas* in HP soil confirmed this result (Supplemental Figure 3C). In addition, the abundance of culturable *Pseudomonas* was 103% higher in *OsPHR2* OE roots than in WT roots in LP soil (Supplemental Figure 4). *Pseudomonas* generally shows high P-solubilization ability (Alori et al., 2017). When applied to P rock fertilizer, *Pseudomonas plecoglossicida* exhibited increased activities of acid phosphatase, alkaline phosphatase, and phytase (Chen and Liu, 2019). Thus, we focused on the OsPHR2-regulated Pseudomonadaceae for subsequent studies of P uptake.

Role of *Pseudomonas* sp. (P6) in rice phosphorus uptake

To further assess the role of Pseudomonadaceae in phosphorus uptake, 10 strains were isolated and obtained from root samples of *OsPHR2* OE rice (Figure 3A). All strains had the capacity for phosphate solubilization, and strain P6 showed the highest phosphate-solubilization ability (phosphate solubilization, 3.54; Figures 3A and 3B). To confirm bacterial phosphate solubilization ability, we used NBRIP (National Botanical Research Institute phosphate growth medium) liquid medium containing $Ca_3(PO_4)_2$. After 6 days of cultivation, the available phosphorus in the medium inoculated with P6 was 127.3 mg L^{-1}, which was the highest concentration among the 10 candidate isolates (Figures 3C and 3D). Thus, strain P6 (*Pseudomonas* sp.) was selected for further analysis.

OsPHR2 OE rice increased abundance of succinic acid and methylmalonic acid for promoting the growth of *Pseudomonas* sp. (P6)

To determine how OsPHR2 alters the root microbiota, we performed metabolite analysis of root exudates. Compared with the WT, *OsPHR2* OE rice had 93 upregulated metabolites and 32 downregulated metabolites under HP conditions (Figure 4A). Kyoto Encyclopedia of Genes and Genomes analysis identified multiple metabolic pathways that were enriched in the differentially abundant metabolites (Figure 4B). Previously, the target gene *OsHAD1*, which is directly regulated by *OsPHR2*, was found to significantly enhance organic acid accumulation in root exudates (Pandey et al., 2017). We therefore speculated that *OsPHR2* might alter organic acid accumulation in root exudates. Further analysis revealed that the relative abundance of succinic acid and methylmalonic acid was 2.6- and 3.5-fold higher in *OsPHR2* OE exudates than in WT exudates (Figures 4C–4E), whereas the abundance of lactic acid was significantly lower in *OsPHR2* OE exudates. To determine whether these organic acids could promote P6 growth, we added them individually to the P6 growth medium. Interestingly, succinic acid and methylmalonic acid stimulated the growth of P6, whereas lactic acid inhibited P6 growth (Figure 4F and Supplemental Figure 5).

Inoculation with *Pseudomonas* sp. (P6) promotes rice phosphorus uptake

To verify the ability of P6 to promote rice phosphorus uptake, WT and *OsPHR2* OE plants were grown under HP and LP conditions, with or without P6 inoculation (Figure 5A). P6 inoculation had no significant effect on total P concentrations in shoots or roots of WT or *OsPHR2* OE rice under HP conditions (Figures 5B and 5C). However, under LP conditions, P6 inoculation significantly increased total P concentrations in shoots and roots by 20% and 73% in the WT (Figures 5B and 5C) and by 36% and 104% in *OsPHR2* OE rice compared with uninoculated plants (Figures 5B and 5C). Under HP conditions, total P concentrations in shoots and roots were 53% and 23% greater in *OsPHR2* OE rice than in WT rice in the absence of inoculation (Figures 5B and 5C) and 56% and 19% greater in *OsPHR2* OE rice than in WT rice with P6 inoculation (Figures 5B and 5C). Under LP conditions, there were no significant differences in total P concentrations of shoots and roots between WT and *OsPHR2* OE rice in the absence of inoculation (Figures 5B and 5C), but total P concentrations of shoots and roots were 11.3% and 24.2% higher in *OsPHR2* OE rice than in WT rice with P6 inoculation (Figures 5B and 5C). We next examined the ability of mixed strains isolated from *OsPHR2* OE roots to promote P uptake. Effects of the mixed strains on P uptake were similar to those of strain P6. Under LP conditions, there were no significant differences in total P concentrations of shoots and roots between WT and *OsPHR2* OE plants in the absence of mixed-strain inoculation (Supplemental

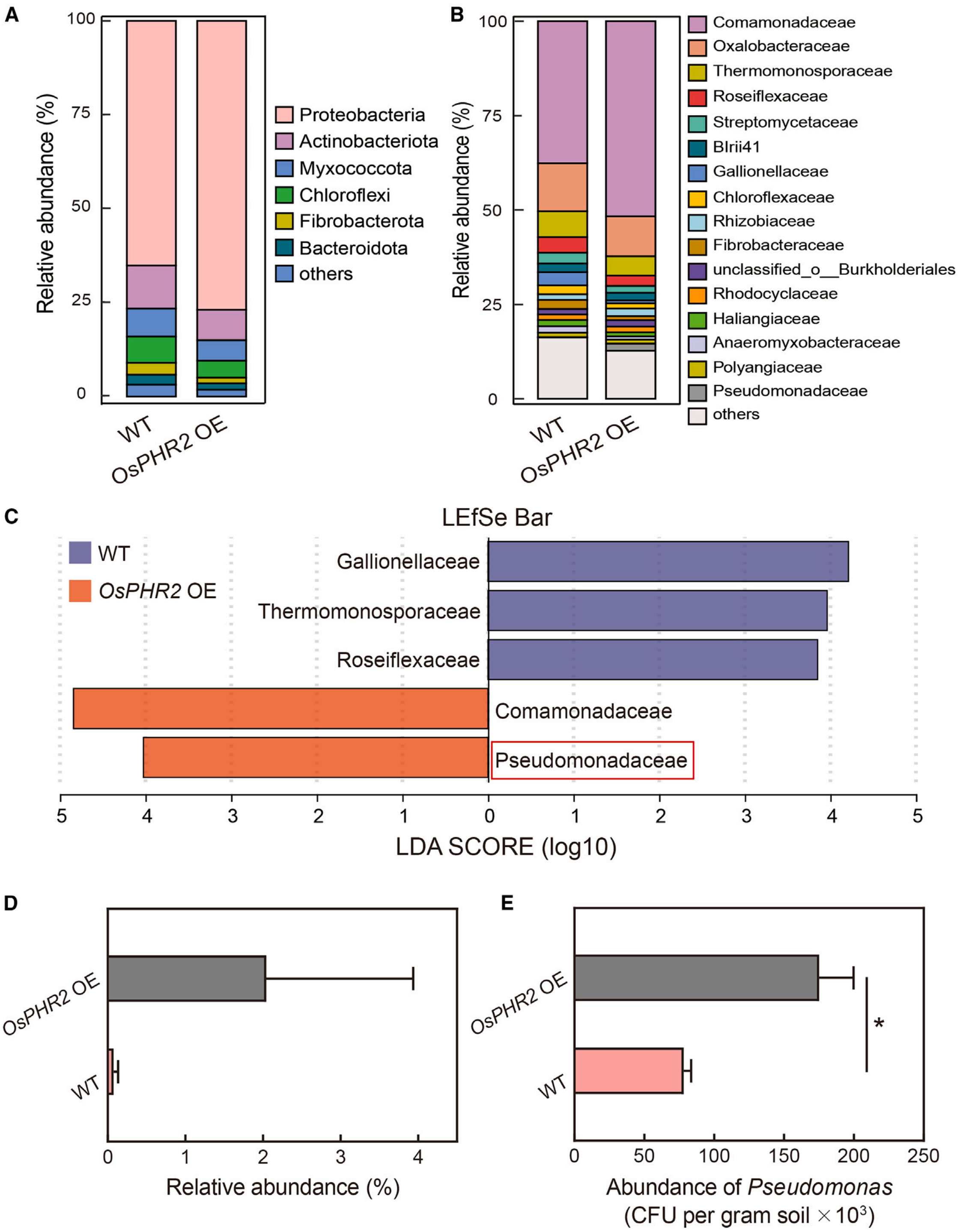

Figure 2. Pseudomonadaceae are associated with phosphorus uptake in *OsPHR2* OE rice.
(A and B) Relative abundance of bacterial taxa at the phylum and family levels from roots of WT and *OsPHR2* OE rice under HP conditions.
(C) Analysis of linear discriminant analysis effect size (LEfSe) of bacterial taxa with significant differences in abundance between WT and *OsPHR2* OE roots. Linear discriminant analysis score $\geq$ 3.8.
(D) Relative abundance of Pseudomonadaceae in WT and *OsPHR2* OE roots. Data are means ± SD (n = 3).
(E) Abundance of culturable *Pseudomonas* in roots of WT and *OsPHR2* OE rice. Data are means ± SD (n = 3).
Asterisks indicate significant differences determined by two-sided Student's t-test ($^*p < 0.05$). See also Supplemental Figure 1.

Figure 6). However, total P concentrations of shoots and roots were significantly higher in *OsPHR2* OE rice than in WT rice upon mixed-strain inoculation (Supplemental Figure 6). To further investigate the mechanism underlying the increased P uptake of *OsPHR2* OE plants under LP + P6 conditions, we measured the expression of Pi transporter genes and the Pi-starvation-induced marker

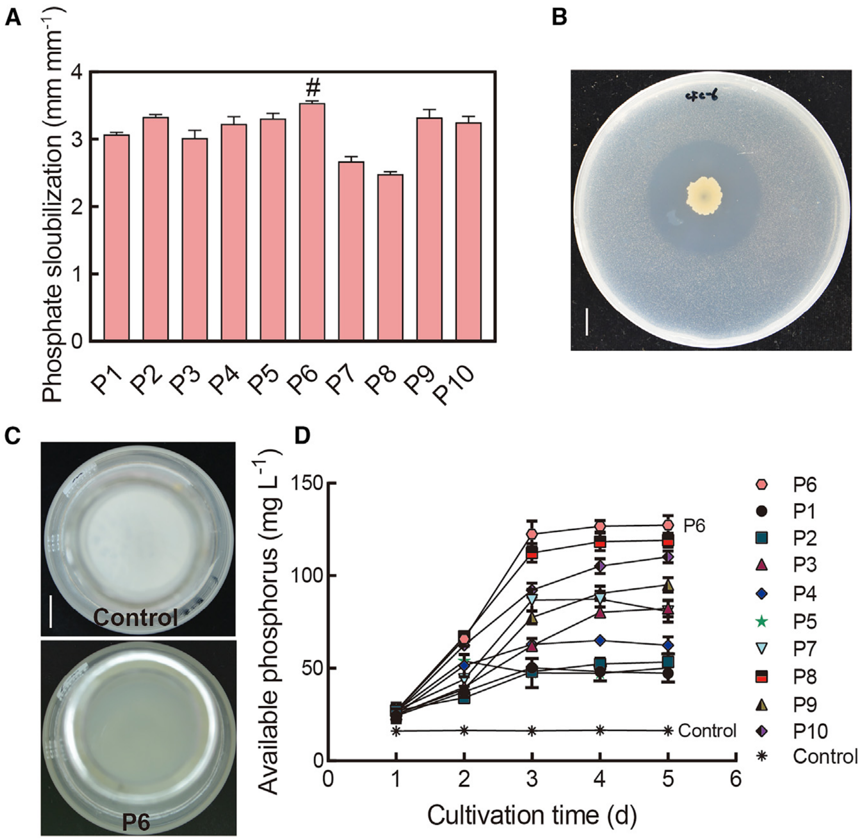

Figure 3. *Pseudomonas* sp. (P6), isolated from the roots of *OsPHR2* OE rice, shows high phosphate solubilization ability.

(A) Phosphate solubilization ability of 10 bacterial strains isolated from *OsPHR2* OE roots and grown on Pikovskaya's agar medium. Phosphate solubilization efficiency was calculated as solubilization zone (mm)/colony diameter (mm). The hash symbol (#) highlights the highest phosphate solubilization efficiency among the 10 isolates. Data are means ± SD (n = 3).

(B) Image of phosphate solubilization by P6. Bar: 1 cm.

(C and D) Available phosphorus concentrations in NBRIP liquid medium. The white sediment visible in the control treatment is $Ca_3(PO_4)_2$, and the gray sediment in the P6-inoculation treatment indicates that some $Ca_3(PO_4)_2$ has been solubilized by P6. Data are means ± SD (n = 3). Bar: 1 cm.

gene *OsIPS1*. Under LP + P6 conditions, the relative expression of *OsIPS1* was 8.68-fold higher in WT plants and 2.22-fold higher in *OsPHR2* OE plants compared with that under LP alone (Supplemental Figure 7). Likewise, expression of the P transporter genes *OsPHT1;1*, *OsPHT1;2*, *OsPHT1;6*, *OsPHT1;8*, and *OsPHT1;10* in WT plants was significantly increased by 67.7%–530.3% under LP + P6 conditions compared with LP alone (Supplemental Figure 7).

DISCUSSION

The microbiota is important for P uptake in *OsPHR2* OE rice

OsPHR2 overexpression results in excessive accumulation of Pi in shoots under Pi-sufficient conditions (Zhou et al., 2008; Wang et al., 2013). In the present study, the shoot phosphorus content of *OsPHR2* OE rice was significantly higher than that of WT rice under HP conditions, consistent with previous studies (Figure 1). PHR proteins are involved in maintaining a beneficial association with the growth-promoting fungus *Colletotrichum tofieldiae* and mycorrhizal fungi under phosphate-starvation conditions in *Arabidopsis* and rice (Hiruma et al., 2016; Shi et al., 2021). The PHR1–RALF–FERONIA axis can shift the root microbiota to alleviate phosphate starvation by regulating the expression of PHR genes (Tang et al., 2022). Here, the toxicity phenotype of *OsPHR2* OE rice was alleviated by growth on sterilized soil (Figure 1A), implying that the soil microbiota may be involved in the enhanced P uptake of *OsPHR2.* In addition, the phosphorus content of *OsPHR2* OE rice was much lower on sterilized soil than on natural soil (Figure 1D), confirming the important effect of microbiota on *OsPHR2*-modulated P accumulation. These results suggest that the microbiota is important for P uptake in *OsPHR2* OE rice.

Pseudomonas promotes rice P uptake, which is associated with phosphate solubilization and P-starvation-induced gene regulation

Rhizosphere microbes can release plant-available phosphorus, promoting efficient root phosphorus uptake through soil P solubilization (Lambers et al., 2009). In the present study, shoot and root P concentrations were significantly higher in WT and *OsPHR2* OE rice inoculated with P6 than in uninoculated plants under LP conditions (Figure 5). This is probably because *Pseudomonas* is an efficient P solubilizer (Oteino et al., 2015). Bacteria are able to secrete various compounds, such as organic acids and siderophores, to solubilize insoluble soil P through binding of –COOH and –OH to metal cations, exchange of organic compounds and adsorbed P, and acidification of the soil solution (Oburger et al., 2011; Wang et al., 2016; Pastore et al., 2020). Here, the strain P6, isolated from roots of *OsPHR2* OE rice, showed high phosphate-solubilization ability (Figure 3), suggesting that it may be able to secrete compounds to solubilize soil P (Chen and Liu, 2019). Relative expression of *OsPHR2* increased significantly under LP conditions (Zhou et al., 2008). *OsPHR2* OE rice can simulate phosphorus-deficiency signals to regulate P-starvation-induced genes even under HP conditions, which suggests that bacteria isolated from *OsPHR2* OE rice may have the ability to promote P uptake. In the present study, we screened root bacteria specifically recruited by *OsPHR2* under HP conditions (Figure 3A). *Pseudomonas* sp. (P6) showed the highest phosphate-solubilization ability of the 10 isolated strains, suggesting that P6 may play an important role in rice P uptake.

Recently, P-solubilizing bacteria have been shown to regulate some specific *PHT1* genes in response to P-deficient conditions (Liu et al., 2019; Murgese et al., 2020; Srivastava and Srivastava,

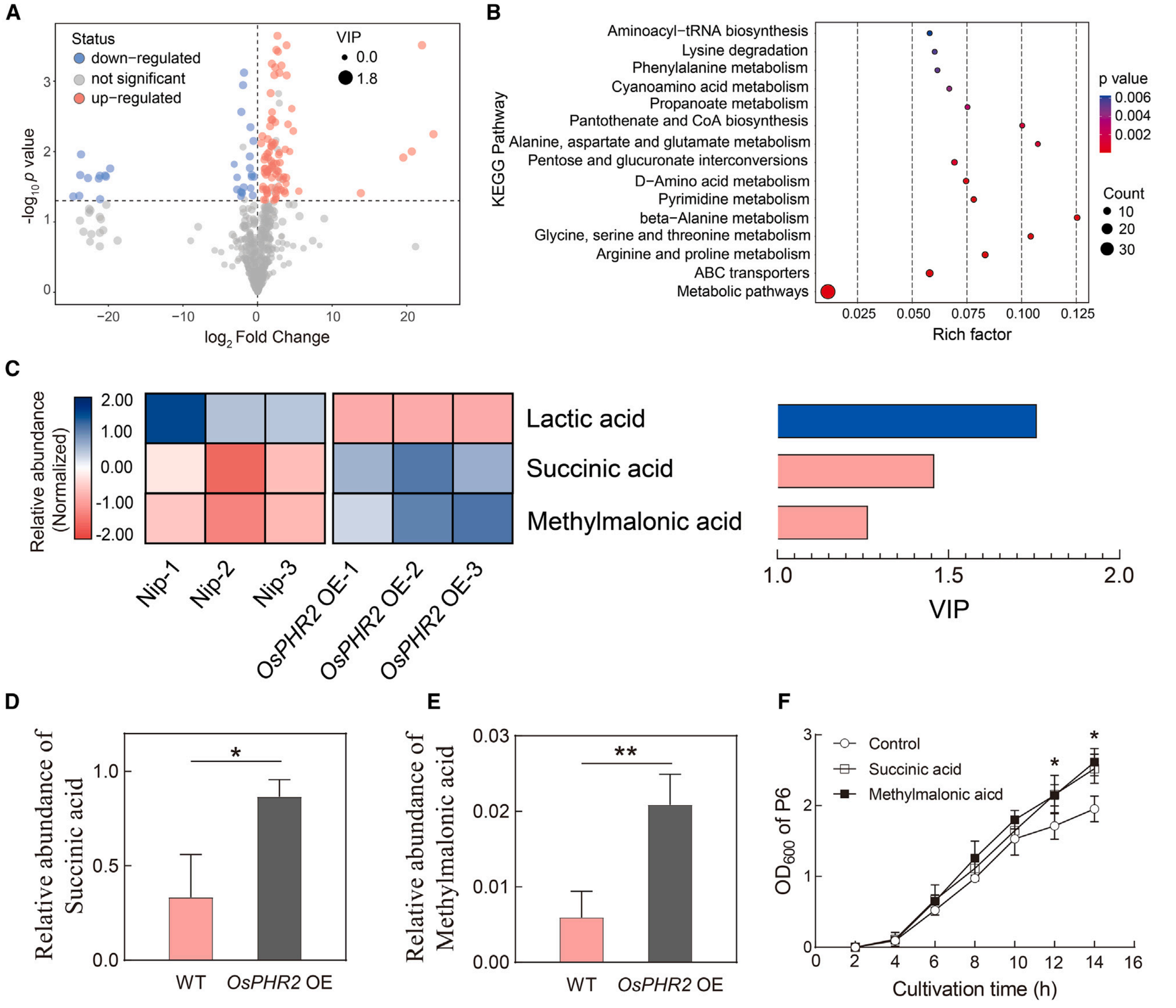

Figure 4. PHR2 enhances the growth of *Pseudomonas* sp. (P6) through biosynthesis of succinic acid and methylmalonic acid in rice roots.

(A) Volcano plot of differentially abundant metabolites between WT and *OsPHR2* OE rice root exudates. VIP, variable importance in projection.

(B) Kyoto Encyclopedia of Genes and Genomes (KEGG) pathways enriched in the differentially abundant metabolites between WT and *OsPHR2* OE rice root exudates.

(C) Heatmap of different organic acids between WT and *OsPHR2* OE rice root exudates. VIP, variable importance in projection.

(D and E) Relative abundance of succinic acid and methylmalonic acid in WT and *OsPHR2* OE rice root exudates. Data are means ± SD ($n = 3$). Asterisks indicate significant differences determined by two-sided Student's *t*-test (*$p < 0.05$ and **$p < 0.01$).

(F) Growth of strain P6 with addition of succinic acid or methylmalonic acid. Data are means ± SD ($n = 5$). Asterisks indicate significant differences determined by two-sided Student's *t*-test (*$p < 0.05$).

2020; Tang et al., 2022). *Bacillus subtilis* significantly upregulated *PHT1;1* and *PHT1;4* under LP conditions (Tang et al., 2022). The expression of *PHT1* genes in *A. thaliana* roots was modulated by *Pseudomonas putida* under P-deficient conditions, and the expression of *PHO2* was downregulated (Srivastava and Srivastava, 2020). Here, expression of the P transporter genes *OsPHT1;1*, *OsPHT1;2*, *OsPHT1;6*, *OsPHT1;8*, and *OsPHT1;10* was higher upon P6 inoculation under LP conditions than under LP conditions alone (Supplemental Figure 7), suggesting that P6, isolated from the roots of *OsPHR2*, can also enhance rice P uptake by regulating P transporter genes. These results suggest that *Pseudomonas* can increase rice P uptake through P solubilization and P transporter gene regulation.

OsPHR2 can recruit the native soil bacterium *Pseudomonas* through root organic acid biosynthesis

Plants can exude organic acids, enzymes, and H^+/OH^- to increase plant physiological activity near the source of P in the rhizosphere (George et al., 2002). Plants release up to 25% of carbon to the root for root exudation to attract and nourish soil microbes (van Dam and Bouwmeester,

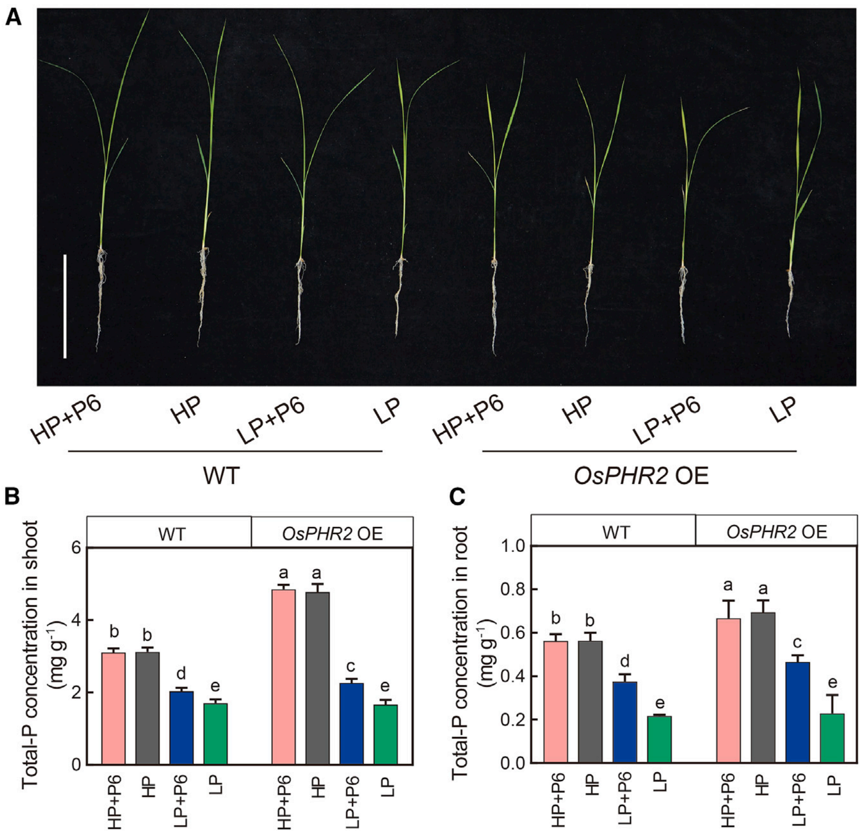

Figure 5. Phosphorus uptake is increased by *Pseudomonas* sp. (P6) inoculation under low-P (LP) conditions.

(A) Phenotypes of WT and *OsPHR2* OE rice grown under HP and LP conditions, with and without P6 inoculation. Bar: 10 cm.

(B and C) Total P concentrations in shoots **(B)** and roots **(C)** of WT and *OsPHR2* OE rice with or without P6 inoculation under HP or LP conditions. Data are means ± SD (n = 5). Bars with different letters are significantly different ($p < 0.05$; ANOVA, Duncan's multiple range test).

2016; Zhalnina et al., 2018). Root exudates can interact with microbes to enhance crop resistance to biotic or abiotic stresses (Xu et al., 2018; Harbort et al., 2020; Wen et al., 2020). Rhizosphere microbial communities can be regulated by root exudates through recruitment of beneficial microorganisms such as phosphate-solubilizing bacteria (Huang et al., 2014). In the present study, the relative abundance of Pseudomonadaceae was greater in roots of *OsPHR2* OE rice than WT rice (Figure 2D and 2E), suggesting that *OsPHR2* may recruit some phosphate-solubilizing bacteria for P uptake. Moreover, the abundance of succinic acid and methylmalonic acid in root exudates was significantly greater in *OsPHR2* OE plants than in WT plants (Figure 4C–4E), consistent with the increased organic acid accumulation in root exudates of rice overexpressing the PHR2-regulated gene *OsHAD1* (Pandey et al., 2017). Colonization by *Bacillus amyloliquefaciens* T-5 can be significantly increased by organic acid exudates of tomato roots (Tan et al., 2013). Here, the growth of strain P6 was significantly promoted by the addition of succinic acid and methylmalonic acid (Figure 4F), consistent with the stimulation of *Enterobacter* growth by cucurbitacin B (Zhong et al., 2022). These results suggest that OsPHR2 can recruit the native soil bacterium *Pseudomonas* for P uptake through root organic acid biosynthesis.

In conclusion, we demonstrated that *OsPHR2* can recruit Pseudomonadaceae through root exudation to promote rice P uptake. *Pseudomonas* can contribute to rice P uptake through P solubilization and regulation of P transporter genes. These results improve our understanding of how OsPHR2 integrates microbiota for P uptake and will help to identify novel research avenues for improving plant resistance to P-deficient conditions.

METHODS

Plant growth conditions

Nipponbare (WT) and *OsPHR2* OE lines were used in this study (Lv et al., 2014). Rice seeds were surface sterilized using 1.5% (v/v) NaClO for 20 min, washed with double-distilled water five times, and grown in 1/2 MS nutrient medium. After 3 days, the seedlings were transplanted into soil pots. All the pot experiments were conducted in the greenhouse with a 14-h light (26°C)/10-h dark (22°C) cycle, 60% (w/w) relative humidity, and a photosynthetic photon flux density of 300 mmol photon m^{-2} s^{-1}. The soil used in this study was collected from a rice experimental field at Fujian Agriculture and Forestry University, Fuzhou City, Fujian Province, China (119°14′E, 26°5′N). The soil chemical factors are listed in Supplemental Table 1. The air-dried soil was sieved through a 4-mm mesh to remove any coarse material and vegetative matter after the addition of mineral nutrients. Five-day-old sterile rice seedlings were then transferred to the soil pots. The pots were distributed in a randomized arrangement in the greenhouse. The sterilized soil was sterilized three times by autoclaving and heat incubation until completely dehydrated (Zhang et al., 2019a, 2019b). After 3 weeks, plant height, biomass, and total P concentration were measured.

Sample collection and DNA extraction for bacterial community analysis

Root samples were collected as described in Zhang et al. (2019a, 2019b). In brief, rice roots were collected and shaken to remove loosely adhering soil, then washed in sterilized water until there was no visible soil. The roots were then placed into a 2-mL tube and stored at −80°C for sequencing. Bulk soil was obtained from pots without plant treatments. For rhizosphere soil, rice roots were shaken to remove loosely adhering soil. Soil attached firmly to the roots was sampled and considered to be the rhizosphere soil (Zhang et al., 2019a, 2019b). DNA was extracted from rice roots, rhizosphere soils, and bulk soils using the Mag-Bind Soil DNA Kit (Omega Bio-Tek). DNA quality and quantity were determined by gel electrophoresis and NanoDrop ONE spectrophotometry (Thermo Scientific, Waltham, MA, USA). Three replicates of rice roots and bulk soil and three replicates of rhizosphere soil of each line were obtained for 16S rRNA high-throughput sequencing.

Bacterial community analysis

The V5-V7 region of the bacterial 16S rRNA gene was amplified using 799F/1193R primers according to Zhang et al. (2019a, 2019b). PCR reactions were run in triplicate in 50-μL mixtures containing 25 μL

Phusion High-Fidelity PCR Master Mix with HF buffer (New England Biolabs), 3 μL primer (10 μM), 10 μL template DNA, 6 μL double-distilled water, and 3 μL dimethyl sulfoxide at 98°C for 30 s; 25 cycles of 98°C for 15 s, 58°C for 15 s, and 72°C for 15 s; and 72°C for 60 s. The PCR products were extracted from 2% agarose gels, purified using an AxyPrep DNA Gel Extraction Kit (Axygen Biosciences, Union City, CA, USA), and quantified using Quanti-Fluor-ST (Promega, Madison, WI, USA). Samples were then sequenced on the MiSeq platform (Illumina, San Diego, CA, USA).

The raw 16S rRNA sequencing reads were quality filtered (i.e., filtered, dereplicated, denoised, merged, and assessed for chimeras) using DADA2 via QIIME2 (Bolyen et al., 2019). Amplicon sequence variants (ASVs) with a frequency less than two were filtered and deleted from the DADA2-generated feature table (Callahan et al., 2016). Mitochondria- and chloroplast-assigned ASVs were deleted from the rice root data. The QIIME2 naive Bayes classifier was used to classify ASVs trained on 99% operational taxonomic units against the SILVA database (v.138) (Quast et al., 2013). LEfSe analysis was used to identify significantly different ($p < 0.05$) taxa between the WT and the overexpression line. The Wilcoxon rank-sum test was used to assess differences in alpha-diversity (based on Chao and Shannon indexes). Differences in bacterial community composition structure were analyzed on the basis of the Bray–Curtis distance using PERMANOVA (Adonis function, 999 permutations) in principal coordinate analysis. For LEfSe analysis, the Kruskal–Wallis rank-sum test was used to detect features with significantly different abundances between assigned families. The sequencing data are available at the Genome Sequence Archive (https://ngdc.cncb.ac.cn/) of the BIG Data Center, Chinese Academy of Sciences, under BioProject accessions PRJCA019859 (root samples) and PRJCA024748 (rhizosphere samples).

Bacterial culture and isolation

The population densities of culturable *Pseudomonas* from fresh root samples were measured using a standard 10-fold dilution plating assay as described in Wei et al. (2019). Three aliquots of dilution were plated on cephaloridine–fucidin–cetrimide *Pseudomonas* semi-selective medium (Mead, 1985). After incubation at 30°C for 2 days, the number of colony-forming units (CFUs) with the colony number on the plates was calculated.

Fresh roots of the overexpression line were selected for isolation of putative *Pseudomonas*. The root suspension was serially diluted and plated on cephaloridine–fucidin–cetrimide *Pseudomonas* semi-selective medium (Tao et al., 2020). *Pseudomonas* colonies were randomly isolated from the selective plates on the basis of colony morphology after incubation at 30°C for 2 days. The interactive isolate was classified by sequencing the 16S rRNA gene with the primers 27F and 1492R.

Phosphate solubilization assay

The assay was performed as described in He et al. (2022). In brief, bacteria were plated at the center of Pikovskaya's agar medium, which consisted of 0.5 g yeast extract, 5.0 g $Ca_3(PO_4)_2$, 10 g dextrose, 0.2 g KCl, 0.5 g $(NH_4)_2SO_4$, 0.0001 g $MnSO_4$, 0.1 g $MgSO_4 \cdot 7H_2O$, 0.0001 g $FeSO_4$, and 15 g agar per liter. After incubation at 30°C for 7 days, phosphate solubilization was measured on the basis of the halo zone surrounding the bacterial colony. The phosphate solubilization efficiency was calculated as solubilization zone (mm)/colony diameter (mm).

NBRIP liquid medium containing 5.0 g $Ca_3(PO4)_2$ was used to confirm the ability of the bacteria to solubilize phosphate (Nautiyal, 1999). A 10-μL aliquot of fresh culture (10^8 CFU/mL) was inoculated into the medium and incubated at 30°C (180 rpm) for 5 days. The solubilized P concentration was tested at 1, 2, 3, 4, and 5 days. Samples (5 mL) of culture were centrifuged at 10 000 rpm for 5 min to obtain cell-free supernatants, and total P concentrations in the supernatants were measured using the molybdate blue method (Murphy and Riley, 1962).

Metabolite analysis of root exudates

Rice seeds were sown and grown in a hydroponic system in HP nutrient solution (1.25 mM NH_4NO_3, 0.3 mM K_2SO_4, 0.3 mM NaH_2PO_4, 1 mM $CaCl_2$, 1 mM $MgSO_4$, 9 μM $MnCl_2$, 0.39 μM Na_2MoO_4, 20 μM H_3BO_4, 0.77 μM $ZnSO_4$, 0.32 μM $CuSO_4$, and 20 μM EDTA-Fe). After growth for 3 weeks, roots of WT and *OsPHR2* OE rice seedlings were pooled and rinsed thoroughly three times with sterilized water. The roots were then transferred to a cylinder filled with sterilized distilled water for exudate collection over 24 h. The collected exudates were passed through a 0.2-μm filter and frozen at −20°C. Then, 30 μL methoxyaminatio hydrochloride was added to the frozen root exudates for analysis. All samples were measured on a gas chromatograph coupled with a time-of-flight mass spectrometer (J&W Scientific, Folsom, CA, USA) at Bio-Tree Technology (Shanghai, China). Three replicates of each sample were obtained for metabolite analyses.

Phosphorus uptake assay with strain inoculation

This experiment was performed using sterilized washed river sand as described in Pang et al. (2018). The sand was air dried and passed through a 2-mm sieve. The basal nutrients (μg g^{-1} dry sand: N 30, S 50, Ca 24, Mg 10, Cu 0.5, Zn 2, Mn 4, B 0.119, Mo 0.4, Fe 5, Cl 23) and a relevant P nutrient were mixed thoroughly into the sand before filling the pots. Nitrogen was supplied as NH_4NO_3 to provide an initial supply after germination. KH_2PO_4 (water-soluble P) or $Ca_3(PO_4)_2$ (insoluble P) was thoroughly mixed into the sand at a rate of 50 μg P (water-soluble P or insoluble P) per gram dry sand before filling the pots. After germination, half of the rice seedlings were inoculated with a P6 suspension (10^8 cell/mL), and the remaining uninoculated seedlings were treated with sterilized double-distilled water. For the mixed-strain inoculation, a mixed-strain suspension was obtained by inoculating the prepared bacterial suspensions in equal volumes. After 7 days of inoculation, the seedlings were grown for 3 weeks as described in the plant growth conditions section. Total P was measured using the molybdate blue method (Murphy and Riley, 1962).

SUPPLEMENTAL INFORMATION

Supplemental information is available at *Plant Communications Online*.

FUNDING

We are grateful for financial support from the STI 2030-Major Project (2023ZD04072), the National Key Research and Development Program of China (2022YFD1900705), the National Natural Science Foundation of China (32171932 and 42307419), the Fujian Province Natural Science Foundation (2021J01088, 2023J01468, and 2023J02010), and a grant from the Education Department of Fujian Province (JAT220063).

AUTHOR CONTRIBUTIONS

F.X., J.L., and W.X. planned and designed the research. J.L., W.L., Q.Z., L.L., S.C., J.Y., and F.X. conducted most of the experiments. F.X. and J.L. analyzed the data. J.L., F.X., W.X., Q.Z., and Z.W. wrote and revised the manuscript. All authors read and approved the final manuscript.

ACKNOWLEDGMENTS

We thank Prof. Chuanzao Mao (Zhejiang University, China) for *OsPHR2* OE rice seeds. No conflict of interest is declared.

Received: November 21, 2023
Revised: April 2, 2024
Accepted: April 25, 2024
Published: April 29, 2024

REFERENCES

Alori, E.T., Glick, B.R., and Babalola, O.O. (2017). Microbial phosphorus solubilization and its potential for use in sustainable agriculture. Front. Microbiol. **8**:971.

Bolyen, E., Rideout, J.R., Dillon, M.R., Bokulich, N.A., Abnet, C.C., Al-Ghalith, G.A., Alexander, H., Alm, E.J., Arumugam, M., Asnicar, F., et al. (2019). Reproducible, interactive, scalable and extensible microbiome data science using QIIME 2. Nat. Biotechnol. **37**:852–857.

Callahan, B.J., McMurdie, P.J., Rosen, M.J., Han, A.W., Johnson, A.J.A., and Holmes, S.P. (2016). DADA2: High-resolution sample inference from Illumina amplicon data. Nat. Methods **13**:581–583.

Chen, Q., and Liu, S. (2019). Identification and characterization of the phosphate-solubilizing bacterium *Pantoea* sp. S32 in reclamation soil in Shanxi, China. Front. Microbiol. **10**:2171.

Castrillo, G., Teixeira, P.J.P.L., Paredes, S.H., Law, T.F., de Lorenzo, L., Feltcher, M.E., Finkel, O.M., Breakfield, N.W., Mieczkowski, P., Jones, C.D., et al. (2017). Direct integration of phosphate starvation and immunity in response to a root microbiome. Nature **543**:513–518.

George, T., Gregory, P., Robinson, J., and Buresh, R. (2002). Changes in phosphorus concentrations and pH in the rhizosphere of some agroforestry and crop species. Plant Soil **246**:65–73.

Gilbert, N. (2009). Environment: the disappearing nutrient. Nature **461**:716–718.

Harbort, C.J., Hashimoto, M., Inoue, H., Niu, Y., Guan, R., Rombolà, A.D., Kopriva, S., Voges, M.J.E.E.E., Sattely, E.S., Garrido-Oter, R., et al. (2020). Root-Secreted coumarins and the microbiota interact to improve iron nutrition in Arabidopsis. Cell Host Microbe **28**:825–837.e6.

He, D., Singh, S.K., Peng, L., Kaushal, R., Vílchez, J.I., Shao, C., Wu, X., Zheng, S., Morcillo, R.J.L., Paré, P.W., et al. (2022). Flavonoid-attracted Aeromonas sp. from the Arabidopsis root microbiome enhances plant dehydration resistance. ISME J. **16**:2622–2632.

Hiruma, K., Gerlach, N., Sacristán, S., Nakano, R.T., Stéphane, H., Kracher, B., Neumann, U., Ramírez, D., Bucher, M., O'Connell, R.J., et al. (2016). Root endophyte colletotrichum tofieldiae confers plant fitness benefits that are phosphate status dependent. Cell **165**:464–474.

Huang, X.F., Chaparro, J.M., Reardon, K.F., Zhang, R., Shen, Q., and Vivanco, J.M. (2014). Rhizosphere interactions: root exudates, microbes, and microbial communities. Botany **92**:267–275.

Isidra-Arellano, M.C., Delaux, P.M., and Valdés-López, O. (2021). The phosphate starvation response system: its role in the regulation of plant–microbe interactions. Plant Cell Physiol. **62**:392–400.

Johnston, A., Poulton, P., Fixen, P., and Curtin, D. (2014). Phosphorus: its efficient use in agriculture. Adv. Agron. **123**:177–228.

Kant, S., Peng, M., and Rothstein, S.J. (2011). Genetic regulation by NLA and MicroRNA827 for maintaining nitrate-dependent phosphate homeostasis in Arabidopsis. PLoS Genet. **7**, e1002021.

Lambers, H., Mougel, C., Jaillard, B., and Hinsinger, P. (2009). Plant–microbe–soil interactions in the rhizosphere: an evolutionary perspective. Plant Soil **321**:83–115.

Liang, J.L., Liu, J., Jia, P., Yang, T.T., Zeng, Q.W., Zhang, S.C., Liao, B., Shu, W.S., and Li, J.T. (2020). Novel phosphate-solubilizing bacteria enhance soil phosphorus cycling following ecological restoration of land degraded by mining. ISME J. **14**:1600–1613.

Lidbury, I., Borsetto, C., Murphy, A., Bottrill, A., Jones, A., Bending, G., Hammond, J., Chen, Y., Wellington, E., and Scanlan, D. (2020). Niche-adaptation in plant-associated Bacteroidetes favours specialization in organic phosphorus mineralization. ISME J. **15**:1040–1055.

Lin, W.Y., Huang, T.K., and Chiou, T.J. (2013). Nitrogen limitation adaptation, a target of microRNA827, mediates degradation of plasma membrane– localized phosphate transporters to maintain phosphate homeostasis in Arabidopsis. Plant Cell **25**:4061–4074.

Liu, F., Wang, Z., Ren, H., Shen, C., Wu, P., Li, Y., Ling, H.Q., Wu, C., and Lian, X. (2010). *OsSPX1* suppresses the function of *OsPHR2* in the regulation of expression of OsPT2 and phosphate homeostasis in shoots of rice. Plant J. **62**:508–517.

Liu, X., Jiang, X., He, X., Zhao, W., Cao, Y., Guo, T., Li, T., Ni, H., and Tang, X. (2019). Phosphate-solubilizing *Pseudomonas* sp. Strain P34-L promotes wheat growth by colonizing the wheat rhizosphere and improving the wheat root system and soil phosphorus nutritional status. J. Plant Growth Regul. **38**:1314–1324.

Lv, Q., Zhong, Y., Wang, Y., Wang, Z., Zhang, L., Shi, J., Wu, Z., Liu, Y., Mao, C., Yi, K., et al. (2014). SPX4 negatively regulates phosphate signaling and homeostasis through its interaction with PHR2 in rice. Plant Cell **26**:1586–1597.

Mead, G. (1985). Enumeration of pseudomonads using cephaloridine-fucidin-cetrimide agar (CFC). Int. J. Food Microbiol. **2**:21–26.

Miura, K., Rus, A., Sharkhuu, A., Yokoi, S., Karthikeyan, A.S., Raghothama, K.G., Baek, D., Koo, Y.D., Jin, J.B., Bressan, R.A., et al. (2005). The Arabidopsis SUMO E3 ligase SIZ1 controls phosphate deficiency responses. Proc. Natl. Acad. Sci. USA **102**:7760–7765.

Mosimann, C., Oberhänsli, T., Ziegler, D., Nassal, D., Kandeler, E., Boller, T., Mäder, P., and Thonar, C. (2017). Tracing of two *Pseudomonas* strains in the root and rhizoplane of maize, as related to their plant growth-promoting effect in contrasting soils. Front. Microbiol. **7**:2150.

Murgese, P., Santamaria, P., Leoni, B., and Crecchio, C. (2020). Ameliorative effects of PGPB on yield, physiological parameters, and nutrient transporter genes expression in barattiere (*Cucumis melo* L.). J. Soil Sci. Plant Nutr. **20**:784–793.

Murphy, J., and Riley, J. (1962). Modified solution method for determination of phosphate in natural water. Anal. Chim. Acta **27**:31–36.

Nautiyal, C.S. (1999). An efficient microbiological medium growth medium for screening phosphate solubilizing microorganisms. FEMS Microbiol. Lett. **170**:265–270.

Oburger, E., Jones, D.L., and Wenzel, W.W. (2011). Phosphorus saturation and pH differentially regulate the efficiency of organic acid anion-mediated P solubilization mechanisms in soil. Plant Soil **341**:363–382.

Oteino, N., Lally, R.D., Kiwanuka, S., Lloyd, A., Ryan, D., Germaine, K.J., and Dowling, D.N. (2015). Plant growth promotion induced by phosphate solubilizing endophytic *Pseudomonas* isolates. Front. Microbiol. **6**:745.

Pandey, B.K., Mehra, P., Verma, L., Bhadouria, J., and Giri, J. (2017). OsHAD1, a haloacid dehalogenase-like APase, enhances phosphate accumulation. Plant Physiol. **174**:2316–2332.

Pang, J., Bansal, R., Zhao, H., Bohuon, E., Lambers, H., Ryan, M.H., Ranathunge, K., and Siddique, K.H.M. (2018). The carboxylate-releasing phosphorus-mobilizing strategy can be proxied by foliar manganese concentration in a large set of chickpea germplasm under low phosphorus supply. New Phytol. **219**:518–529.

Pastore, G., Kernchen, S., and Spohn, M. (2020). Microbial solubilization of silicon and phosphorus from bedrock in relation to abundance of phosphorus-solubilizing bacteria in temperate forest soils. Soil Biol. Biochem. **151**, 108050.

Quast, C., Pruesse, E., Yilmaz, P., Gerken, J., Schweer, T., Yarza, P., Peplies, J., and Glöckner, F.O. (2013). The SILVA ribosomal RNA gene database project: improved data processing and web-based tools. Nucleic Acids Res. **41**:D590–D596.

Rafi, M., Krishnaveni, M., and Charyulu, P. (2019). Phosphate-solubilizing microorganisms and their emerging role in sustainable agriculture. V. Buddolla, ed. 223–233.

Raghothama, K.G. (1999). Phosphate acquisition. Annu. Rev. Plant Physiol. Plant Mol. Biol. **50**:665–693.

Raymond, N.S., Gómez-Muñoz, B., van der Bom, F.J.T., Nybroe, O., Jensen, L.S., Müller-Stöver, D.S., Oberson, A., and Richardson, A.E. (2021). Phosphate-solubilising microorganisms for improved crop productivity: a critical assessment. New Phytol. **229**:1268–1277.

Ruan, W., Guo, M., Wu, P., and Yi, K. (2017). Phosphate starvation induced *OsPHR4* mediates Pi-signaling and homeostasis in rice. Plant Mol. Biol. **93**:327–340.

Rubio, V., Linhares, F., Solano, R., Martín, A.C., Iglesias, J., Leyva, A., and Paz-Ares, J. (2001). A conserved MYB transcription factor involved in phosphate starvation signaling both in vascular plants and in unicellular algae. Genes Dev. **15**:2122–2133.

Shi, J., Zhao, B., Zheng, S., Zhang, X., Wang, X., Dong, W., Xie, Q., Wang, G., Xiao, Y., Chen, F., et al. (2021). A phosphate starvation response-centered network regulates mycorrhizal symbiosis. Cell **184**:5527–5540.e18.

Soumare, A., Boubekri, K., Lyamlouli, K., Hafidi, M., Ouhdouch, Y., and Kouisni, L. (2021). Efficacy of phosphate solubilizing actinobacteria to improve rock phosphate agronomic effectiveness and plant growth promotion. Rhizosphere **17**, 100284.

Srivastava, S., and Srivastava, S. (2020). Prescience of endogenous regulation in *Arabidopsis thaliana* by *Pseudomonas putida* MTCC 5279 under phosphate starved salinity stress condition. Sci. Rep. **10**:5855.

Tan, H., Barret, M., Mooij, M.J., Rice, O., Morrissey, J.P., Dobson, A., Griffiths, B., and O'Gara, F. (2013). Long-term phosphorus fertilisation increased the diversity of the total bacterial community and the phoD phosphorus mineraliser group in pasture soils. Biol. Fertil. Soils **49**:661–672.

Tang, J., Wu, D., Li, X., Wang, L., Xu, L., Zhang, Y., Xu, F., Liu, H., Xie, Q., Dai, S., et al. (2022). RALFFERONIA shapes the root microbiome to alleviate phosphate starvation. EMBO J. **41**, e109102.

Tao, C., Li, R., Xiong, W., Shen, Z., Liu, S., Wang, B., Ruan, Y., Geisen, S., Shen, Q., and Kowalchuk, G.A. (2020). Bio-organic fertilizers stimulate indigenous soil *Pseudomonas* populations to enhance plant disease suppression. Microbiome **8**:137.

Valverde, A., Burgos, A., Fiscella, T., Rivas, R., Velazquez, E., Rodríguez-Barrueco, C., Cervantes, E., Chamber, M., and Igual, J. (2006). Differential effects of coinoculations with *Pseudomonas jessenii* ps06 (a phosphate-solubilizing bacterium) and *Mesorhizobium ciceri* c-2/2 strains on the growth and seed yield of chickpea under greenhouse and field conditions. Plant Soil **287**:43–50.

van Dam, N.M., and Bouwmeester, H.J. (2016). Metabolomics in the rhizosphere: Tapping into belowground chemical communication. Trends Plant Sci. **21**:256–265.

Wang, D., Xie, Y., Jaisi, D.P., and Jin, Y. (2016). Effects of low-molecular-weight organic acids on the dissolution of hydroxyapatite nanoparticles. Environ. Sci.: Nano **3**:768–779.

Wang, J., Sun, J., Miao, J., Guo, J., Shi, Z., He, M., Chen, Y., Zhao, X., Li, B., Han, F., et al. (2013). A phosphate starvation response regulator *Ta-PHR1* is involved in phosphate signaling and increases grain yield in wheat. Ann. Bot. **111**:1139–1153.

Wei, Z., Gu, Y., Friman, V.P., Kowalchuk, G.A., Xu, Y., Shen, Q., and Jousset, A. (2019). Jousset. Initial soil microbiome composition and functioning predetermine future plant health. Sci. Adv. **5**, eaaw0759.

Wen, T., Yuan, J., He, X., Lin, Y., Huang, Q., and Shen, Q. (2020). Enrichment of beneficial cucumber rhizosphere microbes mediated by organic acid secretion. Hortic. Res. **7**:154.

Xu, L., Naylor, D., Dong, Z., Simmons, T., Pierroz, G., Hixson, K.K., Kim, Y.M., Zink, E.M., Engbrecht, K.M., Wang, Y., et al. (2018). Drought delays development of the sorghum root microbiome and enriches for monoderm bacteria. Proc. Natl. Acad. Sci. USA **115**:E4284–E4293.

Zhalnina, K., Louie, K.B., Hao, Z., Mansoori, N., da Rocha, U.N., Shi, S., Cho, H., Karaoz, U., Loqué, D., Bowen, B.P., et al. (2018). Dynamic root exudate chemistry and microbial substrate preferences drive patterns in rhizosphere microbial community assembly. Nat. Microbiol. **3**:470–480.

Zhang, J., Liu, Y.X., Zhang, N., Hu, B., Jin, T., Xu, H., Qin, Y., Yan, P., Zhang, X., Guo, X., et al. (2019a). *NRT1.1B* is associated with root microbiota composition and nitrogen use in field-grown rice. Nat. Biotechnol. **37**:676–684.

Zhang, Y., Wang, X., Xu, F., Song, T., Du, H., Gui, Y., Xu, M., Cao, Y., Dang, X., Rensing, C., et al. (2019b). Combining irrigation scheme and phosphorous application levels for grain yield and their impacts on rhizosphere microbial communities of two rice varieties in a field trial. J. Agric. Food Chem. **67**:10577–10586.

Zhong, Y., Xun, W., Wang, X., Tian, S., Zhang, Y., Li, D., Zhou, Y., Qin, Y., Zhang, B., Zhao, G., et al. (2022). Root-secreted bitter triterpene modulates the rhizosphere microbiota to improve plant fitness. Nat. Plants **8**:887–896.

Zhou, J., Jiao, F., Wu, Z., Li, Y., Wang, X., He, X., Zhong, W., and Wu, P. (2008). *OsPHR2* is involved in phosphate-starvation signaling and excessive phosphate accumulation in shoots of plants. Plant Physiol. **146**:1673–1686.

Plant Communications, Volume *5*

Supplemental information

OsPHR2-mediated recruitment of Pseudomonadaceae enhances rice phosphorus uptake

Jianping Liu, Weifeng Xu, Qian Zhang, Wencheng Liao, Liang Li, Shu Chen, Jinyong Yang, Zhengrui Wang, and Feiyun Xu

1. Supplementary Tables

Table S1 Soil chemical factors.

Soil component	Organic C (g/kg)	Total N (g/kg)	Total P (g/kg)	Total K (g/kg)	Olsen-P (mg/kg)	Exchangeable K (mg/kg)
	35.6	1.02	0.537	36.3	49.5	92

2. **Supplementary Figures**

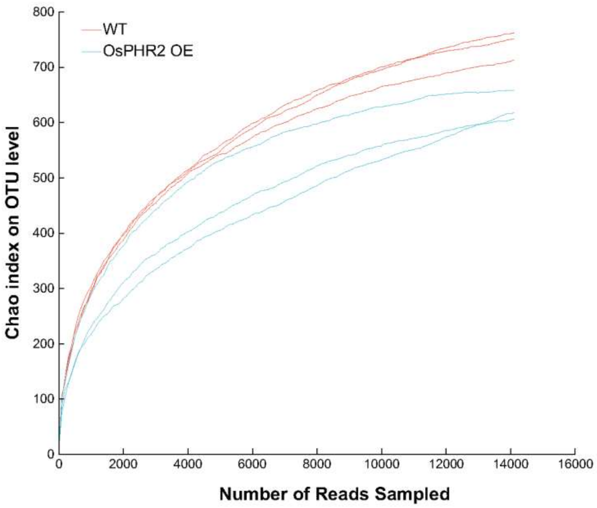

Fig. S1 The gradually flattening rarefaction curves of root samples.

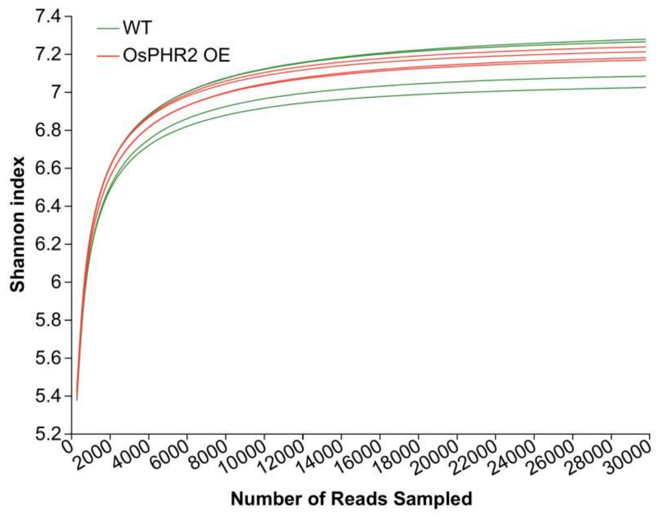

Fig. S2 The gradually flattening rarefaction curves of rhizosphere soil samples.

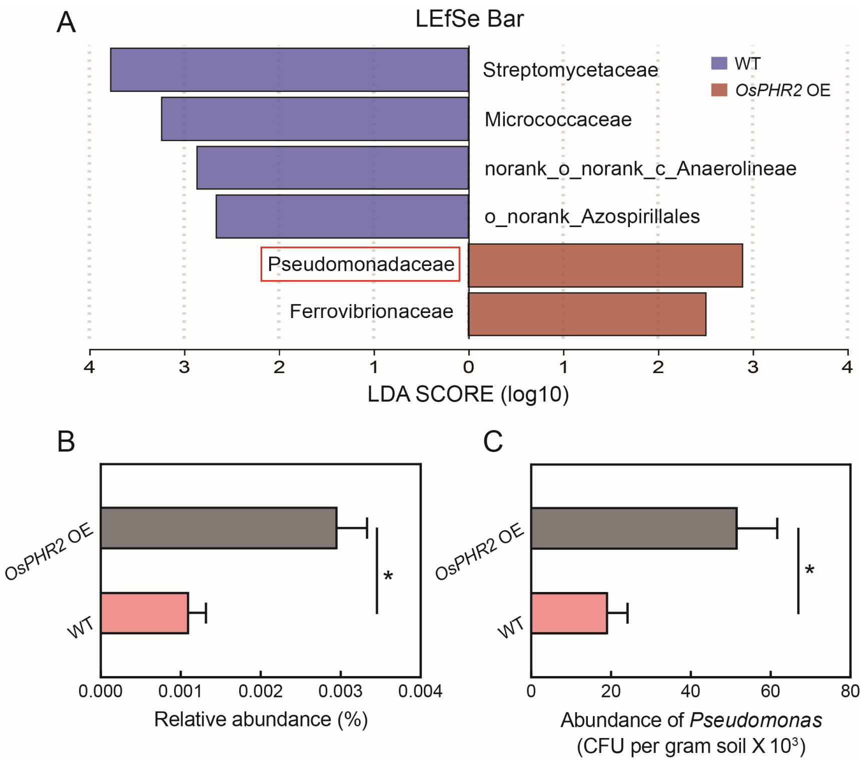

Fig. S3 **A,** Liner discriminant analysis effect size (LEfSe) analysis of bacterial taxon with significant difference in abundances in WT and *OsPHR2* OE rhizosphere soil. Linear discriminant analysis score ≥ 2.5. **B,** Relative abundances of Pseudomonadaceae in WT and *OsPHR2* OE rhizosphere soil. Data are means ± SD (n = 3). **C,** Abundance of cultivable *Pseudomonas* in rhizosphere soil of WT and *OsPHR2* OE rice. Data are means ± SD (n = 3). Asterisks indicate significant difference between soil environments by two-sided Student's t-test (*$p < 0.05$).

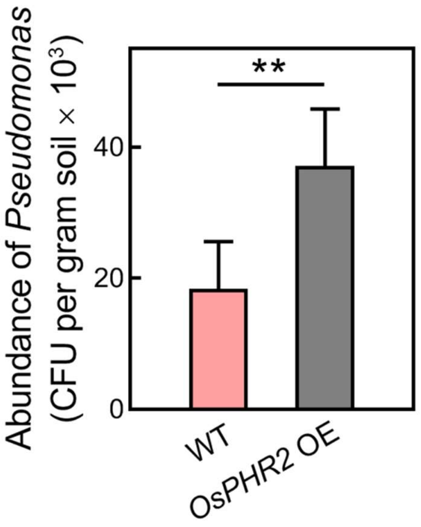

Fig. S4 Abundance of cultivable *Pseudomonas* in root of WT and *OsPHR2* OE rice in low phosphorus soil. Data are means ± SD (n = 6). Asterisks indicate significant difference between soil environments by two-sided Student's t-test (*p < 0.01).

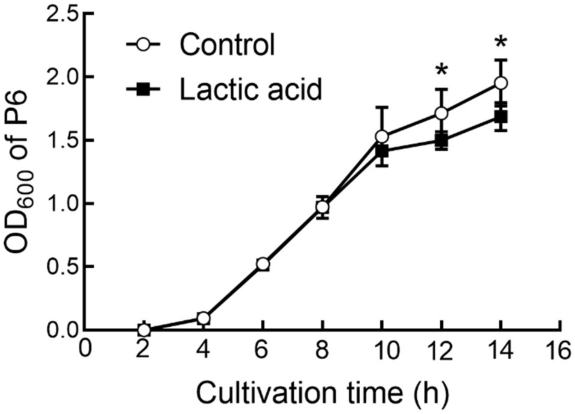

Fig. S5 The growth of strain P6 with addition of lactic aicd. Data are means ± SD (*n* = 5). Asterisks indicate significant difference by two-sided Student's *t*-test (*$p < 0.05$).

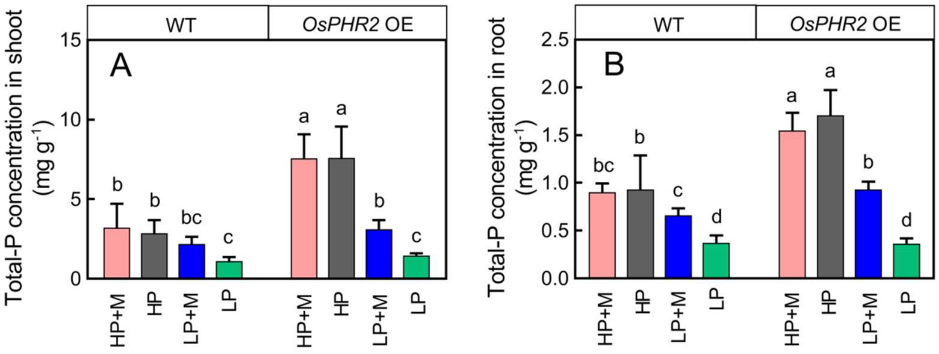

Fig. S6 Shoot and root total P concentration of WT and *OsPHR2* OE rice inoculated with mixed strains under HP (HP+M), not inoculated under HP (HP), inoculated with mixed strains under LP (LP+M), not inoculated under LP (LP). Data are means ± SD (n = 5). Bars with different letters among different treatments indicate significantly different at $p < 0.05$ (ANOVA, Duncan's multiple range test).

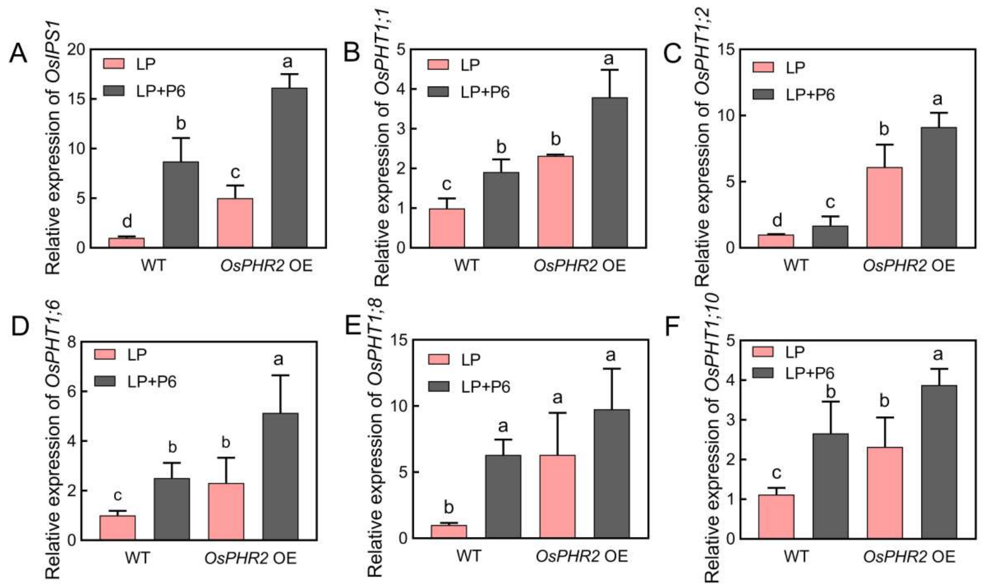

Fig. S7 Relative expression of P transporter genes and P starvation-induced marker gene in WT and *OsPHR2* OE rice inoculated with P6 under LP (LP+P6), not inoculated under LP (LP). Data are means ± SD (n = 3). Bars with different letters among different treatments indicate significantly different at $p < 0.05$ (ANOVA, Duncan's multiple range test).
